# Comparative cytogenetics of *Physalaemus albifrons* and *Physalaemus cuvieri* species groups (Anura, Leptodactylidae)

**DOI:** 10.3897/CompCytogen.v8i2.6414

**Published:** 2014-05-16

**Authors:** Stenio Eder Vittorazzi, Yeda Rumi Serra Douglas Quinderé*, Shirlei Maria Recco-Pimentel, Cristian Tomatis, Diego Baldo, Janaina Reis Ferreira Lima, Juan Martín Ferro, Jucivaldo Dias Lima, Luciana Bolsoni Lourenço

**Affiliations:** 1 Departamento de Biologia Estrutural e Funcional, Instituto de Biologia, Universidade Estadual de Campinas – UNICAMP, 6109, 13083-863, Campinas, São Paulo, Brazil; 2 Laboratorio de Genética Evolutiva, Instituto de Biología Subtropical (CONICET-UNaM), Facultad de Ciencias Exactas Químicas y Naturales, Universidad Nacional de Misiones; Félix de Azara 1552, CPA N3300LQF, Posadas, Misiones, Argentina; 3 Instituto de Pesquisas Científicas e Tecnológicas do Amapá – IEPA, Centro de Pesquisas Zoobotanicas e Geologicas (CPZG), Divisão de Zoologia, Rodovia Juscelino Kubistchek, S/N, Campus da Fazendinha (Distrito da Fazendinha, Macapá, Amapá, Brazil

**Keywords:** Chromosome, NOR, C-banding, heterochromatin, *Physalaemus*

## Abstract

Recently, *Physalaemus albifrons* (Spix, 1824) was relocated from the *Physalaemus cuvieri* group to the same group as *Physalaemus biligonigerus* (Cope, 1861), *Physalaemus marmoratus* (Reinhardt & Lütken, 1862) and *Physalaemus santafecinus* Barrio, 1965. To contribute to the analysis of this proposition, we studied the karyotypes of *Physalaemus albifrons*, *Physalaemus santafecinus* and three species of the *Physalaemus cuvieri* group. The karyotype of *Physalaemus santafecinus* was found to be very similar to those of *Physalaemus biligonigerus* and *Physalaemus marmoratus*, which were previously described. A remarkable characteristic that these three species share is a conspicuous C-band that extends from the pericentromeric region almost to the telomere in the short arm of chromosome 3. This characteristic is not present in the *Physalaemus albifrons* karyotype and could be a synapomorphy of *Physalaemus biligonigerus*, *Physalaemus marmoratus* and *Physalaemus santafecinus*. The karyotype of *Physalaemus santafecinus* is also similar to those of *Physalaemus marmoratus* and *Physalaemus biligonigerus* owing to the presence of several terminal C-bands and the distal localization of the NOR in a small metacentric chromosome. In contrast, the *Physalaemus albifrons* karyotype has no terminal C-bands and its NOR is located interstitially in the long arm of submetacentric chromosome 8. The NOR-bearing chromosome of *Physalaemus albifrons* very closely resembles those found in *Physalaemus albonotatus* (Steindachner, 1864), *Physalaemus cuqui* Lobo, 1993 and some populations of *Physalaemus cuvieri* Fitzinger, 1826. Additionally, the *Physalaemus albifrons* karyotype has an interstitial C-band in chromosome 5 that has been exclusively observed in species of the *Physalaemus cuvieri* group. Therefore, we were not able to identify any chromosomal feature that supports the reallocation of *Physalaemus albifrons*.

## Introduction

Currently, the genus *Physalaemus* Fitzinger, 1826 is classified in the subfamily Leiuperinae Bonaparte, 1850 in the family Leptodatylidae Werner, 1896 (Pyron and Wiens 2011) and is composed of 46 species (Faivovich et al. 2012, Frost 2013). A detailed phylogenetic analysis of the species of *Physalaemus* is not yet available but some supraspecific groupings have been proposed. Lynch (1970) recognized four species groups: *Physalaemus pustulosus*, *Physalaemus biligonigerus*, *Physalaemus cuvieri* and *Physalaemus signifier*, which was followed until recently. Based on a phenetic analysis of morphometric characters, Nascimento et al. (2005) resurrected *Engystomops* Jiménez de la Espada, 1872 to include the species previously allocated to the *Physalaemus pustulosus* group (*sensu* Lynch, 1970), resurrected *Eupemphix* Steindachner,1863 for the single species *Eupemphix nattereri* Steindachner, 1863 (included in the *Physalaemus biligonigerus* group by Lynch, 1970) and recognized seven species groups of *Physalaemus*: *Physalaemus albifrons*, *Physalaemus cuvieri*, *Physalaemus deimaticus*, *Physalaemus gracilis*, *Physalaemus henselii*, *Physalaemus olfersii* and *Physalaemus signifer*. Because *Eupemphix* was paraphyletic with respect to *Physalaemus* in phylogenetic analyses that included eight (Pyron and Wiens 2011) and five (Faivovich et al. 2012) species of *Physalaemus*, Faivovich et al. (2012) proposed that *Eupemphix* is a junior synonym of *Physalaemus*, but did not allocate *Physalaemus nattereri* to any species group. The monophyly of each of the seven species groups of *Physalaemus* proposed by Nascimento et al. (2005) remains to be tested and possible synapomorphies of these groups are still to be recognized (see comments in Borteiro and Kolenc 2007, Tomatis et al. 2009, Vera Candioti et al. 2011).

According to the taxonomic proposal of Nascimento et al. (2005), *Physalaemus albifrons* (Spix, 1824) was removed from the *Physalaemus cuvieri* group (*sensu* Lynch 1970) and grouped together with *Physalaemus biligonigerus* (Cope, 1861), *Physalaemus marmoratus* (Reinhardt & Lütken, 1862) and *Physalaemus santafecinus* Barrio, 1965, three species that were previously allocated to the *Physalaemus biligonigerus* group proposed by Lynch (1970). Interestingly, Lobo (1996) indicated that the species of the *Physalaemus biligonigerus* group (*sensu* Lynch 1970; that included *Physalaemus marmoratus*, as *Physalaemus fuscomaculatus*) shared shovel-shaped metatarsal tubercles with *Physalaemus albifrons*. Otherwise, Vera Candioti et al. (2011) argued that larval oral morphology does not support the reallocation of *Physalaemus albifrons* proposed by Nascimento et al (2005), because the larval oral configuration of *Physalaemus albifrons* is almost identical to that of members of the *Physalaemus cuvieri* species group and differs from that of *Physalaemus biligonigerus*, *Physalaemus santafecinus* and probably *Physalaemus marmoratus*.

Detailed descriptions of the karyotypes of *Physalaemus biligonigerus* and *Physalaemus marmoratus* (as *Physalaemus fuscomaculatus*), which included the identification of the nucleolus organizer regions (NOR) and heterochromatic sites, were already provided (Amaral et al. 2000, Silva et al. 2000). On the other hand, only the chromosome number and morphology are known for *Physalaemus albifrons* (Denaro 1972), and no chromosomal data are available for *Physalaemus santafecinus*.

In the present work, we present a detailed characterization of the karyotype of *Physalaemus albifrons*, describe the karyotype of *Physalaemus santafecinus* and extend the cytogenetic analyses of the *Physalaemus cuvieri* group in order to better characterize the group from which *Physalaemus albifrons* was removed. Our aim is to provide additional evidence that could be used to compare the *Physalaemus albifrons* and *Physalaemus cuvieri* species groups.

## Materials and methods

Specimens of *Physalaemus albifrons*, *Physalaemus santafecinus*, *Physalaemus albonotatus* (Steindachner, 1864), *Physalaemus centralis* Bokermann, 1962 and *Physalaemus cuqui* Lobo, 1993 from different localities in Brazil and Argentina were analyzed. For an unequivocal identification of the species, both morphological and acoustic characteristics were utilized. Each specimen’s locality and voucher number in the scientific collection where it was deposited are provided in Table 1.

**Table 1.** Locality, voucher number and chromosome location of NORs and C-bands of the analyzed specimens. Abbreviations: CH – Chaco province; CT – Corrientes province; MA – Maranhão state; MT - Mato Grosso state; SA – Salta province; SP - São Paulo state; TO - Tocantins state; BC - Departamento de Biologia Celular da UNICAMP, Campinas, SP, Brazil; LGE - Laboratorio de Genética Evolutiva, Facultad de Ciencias Exactas Químicas y Naturales, Universidad Nacional de Misiones, Posadas, Misiones, Argentina; MNRJ - Museu Nacional do Rio de Janeiro, RJ, Brazil; UFMT - Universidade Federal do Mato Grosso, MT, Brazil; ZUEC - Museu de História Natural, Universidade Estadual de Campinas, Campinas, SP, Brazil; p: short arm; q: long arm; int: interstitial; per: pericentromeric; 3cen-per: centromeric band that extend to the short arm. * only one chromosome of the pair. **In the ZUEC 13696 specimen, an additional terminal C-band is present in 2q (see text for details).

**Species**	**Locality**	**Specimens**	**NOR locations**	**Principal non-centromeric C-bands**
*Physalaemus albifrons*	Barreirinhas, MA, Brazil	**7** ♂ (MNRJ 24228, 24230, 24232, ZUEC 12361–3, 17925), **1**♀ (MNRJ 24227)	8q	3cen-per/5p int/8p per/ 9p per
*Physalaemus albonotatus*	Lambari D´Oeste, MT, Brazil (57.4°W, 16.4°S)	**6** ♂ (UFMT 4462, 4466, 4469–72), **1**♀ (UFMT 4465)	8q/9p/9q	2q int/3cen-per/5 int/
*Physalaemus centralis*	Palestina, SP, Brazil (49.2°W, 20.2°S)	**5** ♂ (ZUEC 13689–90, 13692, 3694, 13696)	9q per	2q int**/3cen-per/5p int/ 8q int/9q int/10p per
	Porto Nacional, TO, Brazil (48.6°W, 10.4°S)	**3** ♂ (ZUEC 13373, 13375, 13380)	9q per	2q int/3cen-per/5p int/ 8q int/9q int/10p per
*Physalaemus cuqui*	Near to Rio Piedras, Iruya, SA, Argentina (22°56'S, 64°39'W)	**1**♀ (LGE 6567)	3p*/8q/9p/9q	2q int/3cen-per/5p int
	Taco Pozo, CH, Argentina (25°34'S, 63°09'W)	**2** ♂ (LGE 1635–6)	8q/9p/9q	2q int/3cen-per/5p int
	Aguas Blancas, SA, Argentina (22°43'S, 64°22'W)	**1**♀ (LGE 6568)	8q/9p/9q	-
	Metán, SA, Argentina (25°06'S, 65°03'W)	**1**♀ (LGE 6569)	8q*/9p/9q	2q int/3cen-per/5p int
	Pichanal, SA, Argentina (25°24'S, 64°09'W)	**1**♂ (LGE 6570)	8q/9p/9q	2q int/3cen-per/5p int
*Physalaemus santafecinus*	Ituzaingó, CT, Argentina (27°31'S, 56°40'W	**6** ♂ (LGE 077–8, 083–4, 087–8)	9q	1p per/1q int/2p per/3p/8p per/7q per/terminal in all chromosomes

Metaphase chromosome spreads were obtained from cell suspensions of the intestine and testes of animals pre-treated with colchicine (2%) for at least 4 hours (according to Schmid et al. 2010, or adapted from King and Rofe 1976). Prior to the removal of the intestine and testes, the animals were deeply anesthetized with lidocaine gel 2%. Chromosomes were conventionally stained with 10% Giemsa and sequentially submitted to C-banding (King 1980) and silver staining by the Ag-NOR method (Howell and Black 1980) or to fluorescence *in situ* hybridization (FISH) (Viegas-Péquinot 1992) with the rDNA probe HM 123 (Meunier-Rotival et al. 1979). C-banded metaphases from *Physalaemus albifrons* were also stained with DAPI (0.5 mg/mL). For each species, at least 10 metaphases that were submitted to each technique were analyzed. Morphometric analyses were done using the MICROMEASURE v3.3 software (Reeves and Tear 2000). The chromosomes were classified according to the criteria proposed by Green and Sessions (1991).

## Results

All of the analyzed individuals had a diploid complement of 22 chromosomes. By comparing all of the karyotypes of *Physalaemus* to each other, we noted a high interspecific similarity for the first seven chromosome pairs, and the homeology of these chromosomes could be inferred. Therefore, in each karyotype presented here, these chromosomes were ordered in such a way that their numbers could reflect these homology hypotheses even when their sizes suggested a different numbering. However, the smallest chromosomes (pairs 8–11) varied significantly among the species analyzed, and were numbered only by chromosome size. A detailed description of the karyotype of each species is presented below and the Appendix (Additional file 1) present all the karyotypes arranged together. Table 1 summarizes the data on NORs and non-centromeric C-bands.

### Physalaemus albifrons

**Figure 1.**
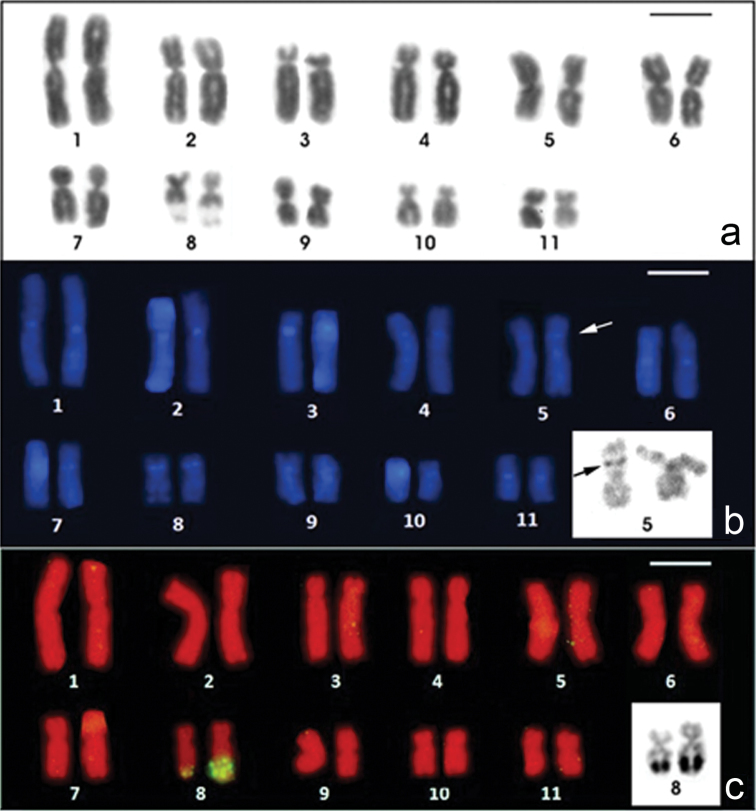
Karyotypes of *Physalaemus albifrons* after Giemsa-staining (**a**) C-banding followed by DAPI-staining (**b**) and in situ hybridization with a nucleolar rDNA probe (**c**). In **b** the inset shows the C-banded chromosome pair 5 stained with Giemsa; in **c** the inset shows the NOR-bearing chromosome pair 8 after silver staining. Arrows in **b** point the interstitial C-band in 5p. Bar=10mm.

The *Physalaemus albifrons* chromosomes were classified as metacentric (pairs 1, 2, 5, 6, 9 and 11), submetacentric (pairs 4, 7, 8 and 10) or subtelocentric (pair 3, which is at the threshold between submetacentric and subtelocentric classifications) (Fig. 1a; Table 2). C-banding followed by DAPI staining detected all of the centromeric regions and an interstitial heterochromatic band in the short arm of chromosome 5 as well as pericentromeric bands in the short arm of chromosomes 8 and 9 (Fig. 1b). The Giemsa stained C-banded metaphases showed this same pattern, but after DAPI staining, the bands could be more easily visualized. Chromosomes 3 and 4 were very similar, but chromosome 3 had a slightly smaller centromeric index and a strong centromeric C-band, which extended to the short arm (Fig. 1b; Table 2).

**Table 2.** Morphometric parameters of the *Physalaemus albifrons*, *Physalaemus albonotatus*, *Physalaemus centralis*, *Physalaemus cuqui* and *Physalaemus santafecinus* karyotypes. The measurements were based on 10 metaphases of each species. CN: chromosome number; CI: centromeric index; SD: standard deviation; RL: relative length. CC: chromosome classification; m: metacentric; sm: submetacentric; st: subtelocentric. *^1^Chromosomes were numbered in order to reflect our hypotheses of homeology for the *Physalaemus* chromosomes even when their sizes suggested a different numbering. *^2^Value at the threshold between submetacentric and subtelocentric classifications.

*Physalaemus albifrons*											
**CN**	**1**	**2**	**3**	**4**	**5**	**6**	**7**	**8**	**9**	**10**	**11**
**CI ± SD**	0.47 ± 0.03	0.40 ± 0.04	0.24 ± 0.02	0.29 ± 0.02	0.46 ± 0.02	0.44 ± 0.04	0.36 ± 0.02	0.33 ± 0.04	0.43 ± 0.04	0.28 ± 0.04	0.45 ± 0.04
**RL**(%)	14.68	12.15	10.06*^1^	10.64*^1^	9.68	9.43	8.27	7.17	6.76	5.94	5.88
**CC**	m	m	st*	sm	m	m	sm	sm	m	sm	m
*Physalaemus albonotatus*											
**CN**	**1**	**2**	**3**	**4**	**5**	**6**	**7**	**8**	**9**	**10**	**11**
**CI ± SD**	0.46 ± 0.03	0.45 ± 0.04	0.24 ± 0.02	0.33 ± 0.02	0.46 ± 0.03	0.43 ± 0.04	0.36 ± 0.07	0.39 ± 0.04	0.44 ± 0.03	0.42 ± 0.03	0.46 ± 0.03
**RL**(%)	13.87	12.18	10.00*^1^	10.42*^1^	9.61	9.48	8.31	7.32	7.05	5.98	5.78
**CC**	m	m	st*	sm	m	m	sm	m	m	m	m
*Physalaemus centralis*											
**CN**	**1**	**2**	**3**	**4**	**5**	**6**	**7**	**8**	**9**	**10**	**11**
**CI ± SD**	0.47 ± 0.01	0.39 ± 0.01	0.26 ± 0.02	0.30 ± 0.03	0.46 ± 0.03	0.43 ± 0.03	0.35 ± 0.03	0.42 ± 0.05	0.45 ± 0.01	0.40 ± 0.02	0.40 ± 0.04
**RL**(%)	13.82	12.24	10.07*^1^	10.26*^1^	10.03	9.36	7.99	7.27	7.12	6.31	5.52
**CC**	m	m	sm	sm	m	m	sm	m	m	m	m
*Physalaemus cuqui*											
**CN**	**1**	**2**	**3**	**4**	**5**	**6**	**7**	**8**	**9**	**10**	**11**
**CI ± SD**	0.47 ± 0.02	0.41 ± 0.03	0.24 ± 0.05	0.30 ± 0.03	0.44 ± 0.03	0.41 ± 0.02	0.34 ± 0.03	0.42 ± 0.01	0.42 ± 0.06	0.38 ± 0.01	0.43 ± 0.03
**RL**(%)	14.53	13.57	10.0*^1^	10.36*^1^	9.93	9.49	8.39	7.08	6.07	5.40	5.19
**CC**	m	m	st*	sm	m	m	sm	m	m	m	m
*Physalaemus santafecinus*											
**CN**	**1**	**2**	**3**	**4**	**5**	**6**	**7**	**8**	**9**	**10**	**11**
**CI ± SD**	0.46 ± 0.02	0.40 ± 0.01	0.39 ± 0.02	0.27 ± 0.02	0.46 ± 0.02	0.43 ± 0.01	0.32 ± 0.02	0.39 ± 0.03	0.47 ± 0.01	0.43 ± 0.03	0.43 ± 0.04
**RL**(%)	14.11	13.21	12.34	10.88	10.27	9.67	8.60	5.68	5.41	5.35	4.47
**CC**	m	m	m	sm	m	m	sm	m	m	m	m

The NORs were located distally in the long arm of chromosome 8 (Fig. 1c) and coincided with the secondary constrictions that were observed in Giemsa-stained metaphases (Fig. 1a). In three specimens (ZUEC 17925, ZUEC 12363 and MNRJ 24224), a size heteromorphism was observed between the homologous NORs by FISH with an rDNA probe (Fig. 1c) and by silver staining (Fig. 1c - inset). In two specimens (MNRJ 24230 and 24232), the NOR-bearing homologous chromosomes 8 were homomorphic. For the remaining specimens, we were not able to determine if a NOR size heteromorphism was present.

### Physalaemus albonotatus

**Figure 2.**
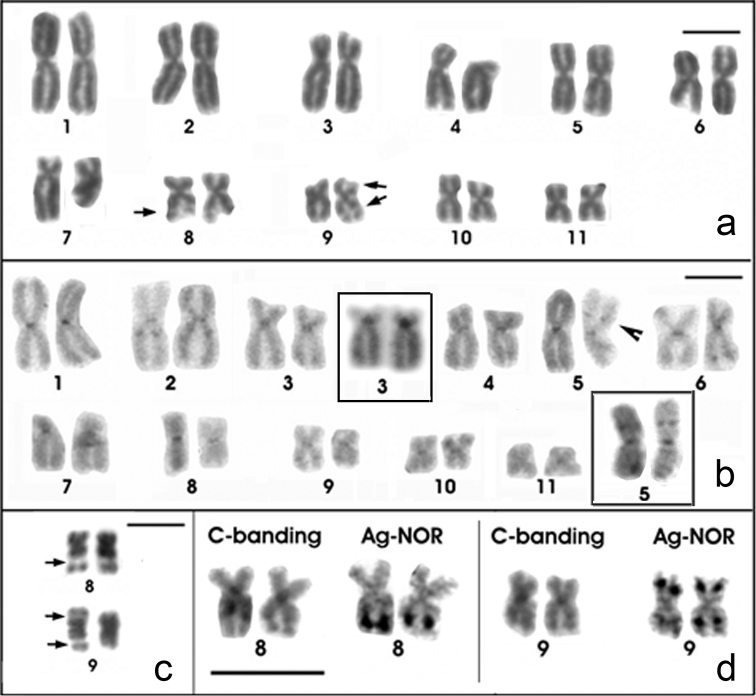
Giemsa-stained (**a**) and C-banded (**b**) karyotypes of *Physalaemus albonotatus*. In the insets in **b** C-banded chromosome pairs 3 and 5, showing evident pericentromeric and interstitial bands, respectively **c** NOR-bearing chromosome pairs of *Physalaemus albonotatus* stained with Giemsa. Arrows in **a** and **c** indicate secondary constrictions of the NORs. Arrowhead in **b** indicates the C-band in chromosome 5 **d** NOR-bearing chromosome pairs of one specimen of *Physalaemus albonotatus* sequentially submitted to the C-banding and the Ag-NOR methods. Note the NOR adjacent to an interstitial C-band in pair 8 and the NORs coincident with faint C-bands in pair 9. Bar=10mm.

The *Physalaemus albonotatus* chromosomes were classified as metacentric (pairs 1, 2, 5, 6, 8, 9, 10 and 11), submetacentric (pairs 4 and 7) or subtelocentric (pair 3, which is at the threshold between submetacentric and subtelocentric classifications) (Fig. 2a; Table 2). Curiously, chromosome 5 was larger than chromosomes 3 and 4 in some of the analyzed metaphases (as seen in Figure 2b). Heterochromatin was detected in the centromeres of all chromosomes and interstitially in the long arm of chromosome 2 and in the metacentric chromosome 5 (Fig. 2b). Only two C-banded chromosome pairs 5 were good enough to be measured. Therefore, we tentatively assigned the interstitial C-band of chromosome 5 to its short arm, but further analyses are necessary to test this hypothesis. Chromosomes 3 and 4 were very similar, but chromosome 3 had a slightly smaller centromeric index and a strong centromeric C-band, which extended to the short arm (Fig. 2b - inset; Table 2).

Silver staining detected NORs distally in the long arm of chromosome 8 adjacent to a faint C-band and in both arms of chromosome 9 (Fig. 2d). The NOR in the long arm of chromosome 9 apparently coincided with a C-band (Fig. 2d). All of these NORs could be seen as secondary constrictions in Giemsa-stained metaphases (Figs. 2a and 2c).

### Physalaemus centralis

**Figure 3.**
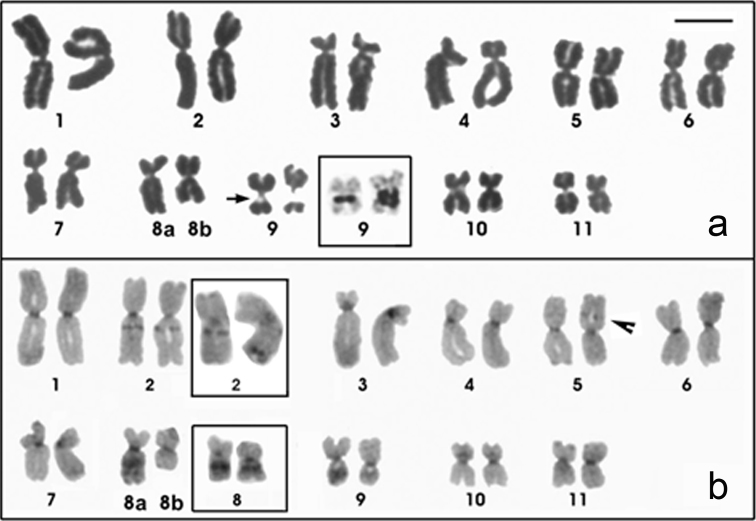
Giemsa-stained (**a**) and C-banded (**b**) karyotypes of *Physalaemus centralis*. In **a** an arrow indicates the secondary constriction of the NOR and the inset shows the NOR-bearing chromosome 9 after silver staining. Note the NOR size heteromorphism. In **b** the arrowhead indicates the C-band in 5p and the insets show the heteromorphic pair 2 and the homomorphic pair 8 of the ZUEC 13696 specimen. Note the conspicuous interstitial heterochromatin in the long arm of chromosome pair 8. Bar=10mm.

The *Physalaemus centralis* chromosomes were classified as metacentric (pairs 1, 2, 5, 6, 8, 9, 10 and 11) or submetacentric (pairs 3, 4, 7 and 8) (Fig. 3a; Table 2). A secondary constriction was detected in the pericentromeric region of the long arm of chromosome 9 and coincided with the NOR that was recognized by silver staining (Fig. 3a - inset). A NOR size heteromorphism was observed in all of the *Physalaemus centralis* specimens analyzed. C-bands were present interstitially in the long arm of chromosome 2, in the short arm of chromosome 5, in the long arms of chromosomes 8 and 9, in the pericentromeric region of the short arm of chromosome 10, and in all of the centromeres (Fig. 3b). Chromosomes 3 and 4 were very similar, but chromosome 3 had a slightly smaller centromeric index and a strong centromeric C-band, which extended to the short arm (Fig. 3b; Table 2).

In three specimens, a heteromorphic chromosome pair 8 composed of homologues with different morphologies and C-banding patterns was observed (Figs. 3a and 3b). While one chromosome 8 showed a conspicuous interstitial C-band that sometimes could be seen as two heterochromatic blocks (chromosome 8a in Fig. 3b), its homologue had no observable interstitial heterochromatic block (Fig. 3b). In the ZUEC 13696 specimen, the pericentromeric C-bands in the long arms of the homologous chromosomes 2 were heteromorphic in size. Additionally, the homologue that had the smaller pericentromeric C-band also had an additional and conspicuous terminal C-band in the long arm (Fig. 3b - inset).

### Physalaemus cuqui

**Figure 4.**
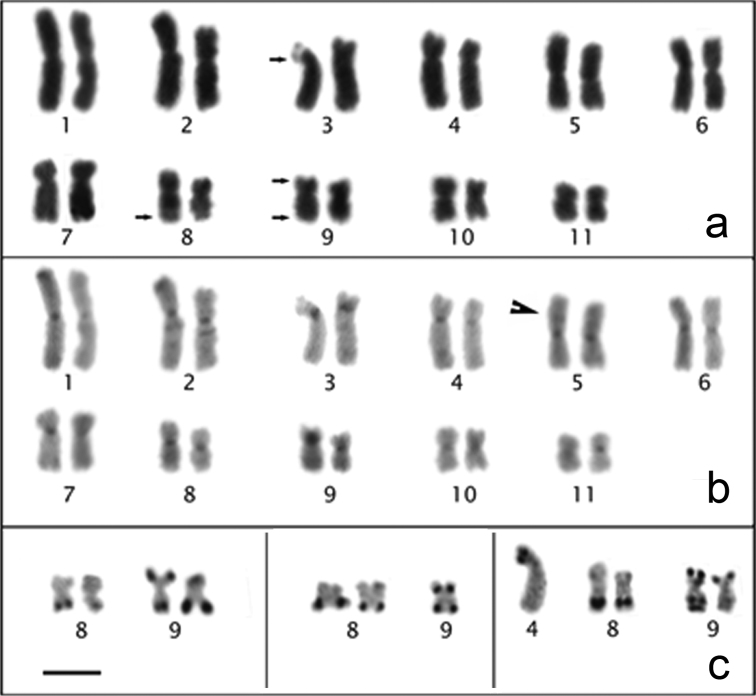
Giemsa-stained (**a**) and C-banded (**b**) karyotypes of *Physalaemus cuqui*. In **a** the arrows indicate the secondary constriction of the NOR. In **b** arrowhead indicates the C-band in 5p **c** Variability in the NOR-bearing chromosome pairs of three specimens of *Physalaemus cuqui*. Bar=10mm.

The *Physalaemus cuqui* chromosomes were classified as metacentric (pairs 1, 2, 5, 6, 8, 9, 10 and 11), submetacentric (pairs 4 and 7) or subtelocentric (pair 3, which is at the threshold between submetacentric and subtelocentric classifications) (Fig. 4a; Table 2). Heterochromatic bands were observed interstitially in the long arm of chromosome pair 2, in the metacentric chromosome pair 5 and in the centromeric regions of all of the chromosomes (Fig. 4b). Only one C-banded chromosome pair 5 could be measured. Therefore, as well as for *Physalaemus albonotatus*, we tentatively assigned the interstitial C-band of chromosome 5 of *Physalaemus cuqui* to its short arm, but further analyses are necessary to test this hypothesis. Chromosomes 3 and 4 were very similar, but chromosome 3 had a slightly smaller centromeric index and a strong centromeric C-band, which extended to the short arm (Fig. 4b; Table 2).

In three specimens, the Ag-NORs were located in the long arm of chromosome pair 8 and in the short and long arms of chromosome pair 9 (LGE 1635-1636, MLP DB 4973) (Fig. 4c – left), but only one chromosome 9 was silver-stained in the MLP DB 5560 specimen (Fig. 4c – middle). Additionally, one specimen (MLP DB 6480) showed an additional Ag-NOR in the short arm of one chromosome 4 (Fig. 4c – right). These Ag-NORs were coincident with the secondary constrictions visualized in Giemsa-stained metaphases (Fig. 4a).

### Physalaemus santafecinus

**Figure 5.**
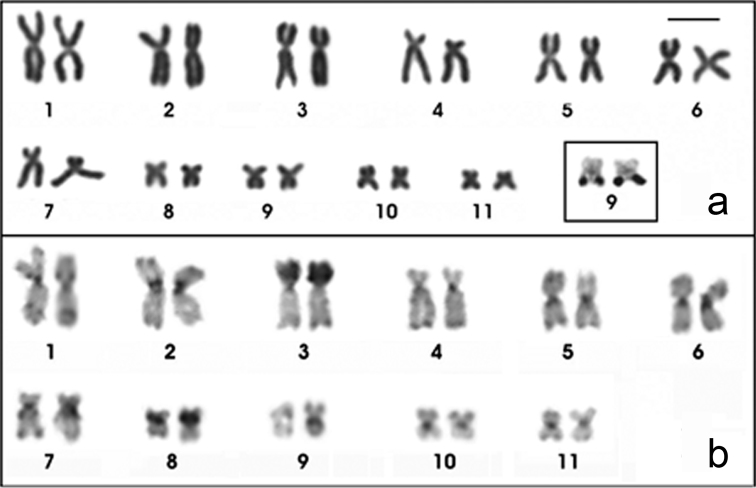
Giemsa-stained (**a**) and C-banded (**b**) karyotypes of *Physalaemus santafecinus*. In **a**, the inset shows the NOR-bearing chromosome 9 after silver staining. Bar=10mm.

The *Physalaemus santafecinus* chromosomes were classified as metacentric (pairs 1, 2, 3, 5, 6, 8, 9, 10 and 11) or submetacentric (pairs 4 and 7) (Fig. 5a; Table 2). The NORs were located distally in the long arm of chromosome 9 (Fig. 5a - inset). C-bands were detected in all the centromeric regions. Additionally, pericentromeric C-bands were present in the short arms of chromosomes 1 and 2 and in the short arm of chromosome 8. Small C-bands were also detected proximally in the long arms of chromosomes 4 and 7 and distally in the long arm of chromosome 1. A conspicuous C-band was observed in the short arm of chromosome 3, which was almost entirely heterochromatic. Terminal faint C-bands could be seen in all of the chromosomes (Fig. 5b). When the Ag-NOR method was performed on C-banded metaphases, we could undoubtedly recognize the chromosome 9 as the NOR-bearing chromosome while chromosomes 8 had strong pericentromeric C-bands (data not shown).

## Discussion

To date, 23 of the 46 species of *Physalaemus* were karyotyped and all of them have 2n=22 (Beçak 1968, Beçak et al. 1970, Denaro 1972, De Lucca et al. 1974, Silva et al. 1999, Silva et al. 2000, Amaral et al. 2000, Lourenço et al. 2006, Ananias et al. 2007, Tomatis et al. 2009, Milani et al. 2010 – included *Physalaemus feioi* Cassini et al., 2010 as *Physalaemus olfersii* (Lichtenstein & Martens, 1856), Nascimento et al. 2010, Provete et al. 2012). Interestingly, two distinct fundamental numbers (FN) can be recognized among the karyotypes of *Physalaemus* species. The five species of the *Physalaemus signifer* group already karyotyped have FN=42 and a telocentric chromosome 11 [see karyotype of *Physalaemus signifer* (Girard, 1853) in De Lucca et al. (1974), *Physalaemus crombiei* Heyer & Wolf, 1989 and *Physalaemus spiniger* (Miranda-Ribeiro, 1926) karyotypes in Silva et al. (2000), and a reference to the *Physalaemus atlanticus* Haddad and Sazima, 2004 and *Physalaemus moreirae* (Miranda-Ribeiro, 1937) karyotypes in the discussion of Ananias et al. (2007)], as does *Physalaemus nattereri* (Beçak 1968, Lourenço et al. 2006, Ananias et al. 2007) and *Physalaemus fernandezae* (Müller, 1926) (Tomatis et al. 2009). The karyotypes of the remaining species of *Physalaemus*, including the species of the *Physalaemus cuvieri* and the *Physalaemus albifrons* groups that we focused on in our present investigation, have FN=44 and a biarmed chromosome 11. Considering the close phylogenetic relationship inferred for *Physalaemus nattereri* and *Physalaemus signifer* (Pyron and Wiens 2011, Faivovich et al. 2012, Fouquet et al. 2013), which was the only species of the *Physalaemus signifer* group already included in phylogenetic analyses, it is possible to suppose that the telocentric chromosomes 11 of *Physalaemus nattereri* and *Physalaemus signifer* have the same origin. On the contrary, the similar chromosomes 11 of *Physalaemus fernandezae* and the *Physalaemus signifer* group probably result from a homoplasy (Tomatis et al. 2009).

The karyotype of *Physalaemus santafecinus* described here is very similar in chromosomal size and morphology to those of *Physalaemus biligonigerus*, *Physalaemus marmoratus* and *Physalaemus* sp. aff. *biligonigerus* (Amaral et al. 2000, Silva et al. 2000). The chromosomes classified by Amaral et al. (2000) as 4 and 5 probably correspond to chromosomes 5 and 4, respectively, of the karyotype of *Physalaemus biligonigerus* described by Silva et al. (2000) and of the *Physalaemus santafecinus* karyotype. Such a discrepancy emerges, however, from the use of different criteria for the numeric classification of the chromosomes rather than from a real divergence between the karyotypes.

A remarkable characteristic of the *Physalaemus santafecinus* karyotype that is shared with the karyotypes of *Physalaemus biligonigerus*, *Physalaemus marmoratus* and *Physalaemus* sp. aff. *biligonigerus* is a conspicuous C-block on the short arm of chromosome 3 (3p) (Table 3). This large heterochromatic C-block is not detected in *Physalaemus albifrons* or in any species of *Physalaemus cuvieri* group. Instead, a small C-band pericentromerically located on 3p was already detected in the karyotypes of the species currently allocated to the *Physalaemus cuvieri* group that were already studied by C-banding [i.e., *Physalaemus albifrons*, *Physalaemus albonotatus*, *Physalaemus centralis*, *Physalaemus cuqui* (present work), *Physalaemus ephippifer* (Steindachner, 1864) (Nascimento et al. 2010) and one of the populations of *Physalaemus cuvieri* Fitzinger, 1826 that was studied cytogenetically by Quinderé et al. (2009)]. Although the pericentromeric C-band in 3p of *Physalaemus ephippifer* could be easily observed, it was also much smaller than those observed in *Physalaemus santafecinus*, *Physalaemus biligonigerus*, *Physalaemus marmoratus* and *Physalaemus* sp. aff. *biligonigerus*. In the latter four species, the larger size of this C-band probably explains the larger size of 3p in these karyotypes. A small pericentromeric C-band that extend from the centromere to the short arm of chromosome 3 was also present in *Physalaemus barrioi* Bokermann, 1967 (Provete et al., 2012), *Physalaemus olfersii* and *Physalaemus feioi* (as *Physalaemus olfersii*; Milani et al. 2010), which are the species of *Physalaemus gracilis* group (*Physalaemus barrioi*) and *Physalaemus olfersii* group (*Physalaemus olfersii* and *Physalaemus feioi*) already studied by C-banding.

**Table 3.** Comparison of chromosome 3 of species of *Physalaemus cuvieri* (left column) and P.* albifrons* (right column) groups. Black areas in the ideograms represent C-bands. *^1^Based on Silva et al. (1999) and Quinderé et al. (2009). *^2^Nascimento et al. (2010). *^3^Based on Amaral et al. (2000).

***Physalaemus cuvieri* group (sensu Nascimento et al. 2005)**	**Chromosome 3**		***Physalaemus albifrons* group (sensu Nascimento et al. 2005)**
*Physalaemus albonotatus*	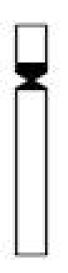	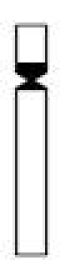	*Physalaemus albifrons*
*Physalaemus centralis*	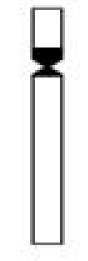	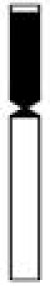 *^3^	*Physalaemus biligonigerus*
*Physalaemus cicada*	No C-banding data	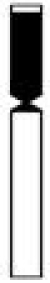 *^3^	*Physalaemus marmoratus* (=*Physalaemus fuscomaculatus*)
*Physalaemus cuqui*	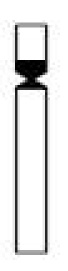	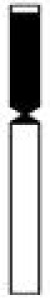	*Physalaemus santafecinus*
*Physalaemus cuvieri*	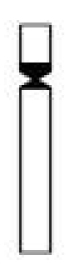 *^1^		
*Physalaemus ephippifer*	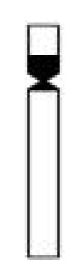 *^2^		
*Physalaemus erikae*	No C-banding data		
*Physalaemus fischeri*	No C-banding data		
*Physalaemus kroyeri*	No C-banding data		

Interestingly, a large 3p showing a large C-band was also observed in *Physalaemus nattereri* (Lourenço et al. 2006, Ananias et al. 2007), a species previously allocated to the *Physalaemus biligonigerus* group by Lynch (1970). Although a rigorous phylogenetic analysis of the *Physalaemus* genus is not yet available, in recent phylogenetic inferences *Physalaemus nattereri* was recovered as the sister species of *Physalaemus signifer* and was not closely related to *Physalaemus biligonigerus* (Pyron and Wiens 2011, Faivovich et al. 2012, Fouquet et al. 2013). In this phylogenetic context the most parsimonious hypothesis is to consider the large heterochromatic region in chromosome 3 of *Physalaemus nattereri* to be homoplastic with respect to the large heterochromatic region in chromosome 3 of the *Physalaemus santafecinus*, *Physalaemus biligonigerus*, *Physalaemus marmoratus* and *Physalaemus* sp. aff. *biligonigerus* karyotypes. This hypothesis is particularly plausible if we consider the evolutionary dynamics of satellite DNAs, which are the principal components of heterochromatin (reviewed in Charlesworth et al. 1994). The copy number of satellite DNA repeats can vary dramatically, as they are frequently involved in unequal crossing over and other events as rolling circle replication and conversion-like mechanisms (reviewed in Charlesworth et al. 1994, and in Ugarkovic and Plohl 2002).

On the other hand, the available data do not prevent the large C-band found on 3p of *Physalaemus santafecinus*, *Physalaemus biligonigerus* and *Physalaemus marmoratus* from being a synapomorphy of this group of species, which could have arisen from the amplification of a small C-band. Despite the proposals of Lynch (1970) and Nascimento et al. (2005) disagree with regard to the relationships of these three species with other *Physalaemus* species, the close relationships of *Physalaemus santafecinus*, *Physalaemus biligonigerus* and *Physalaemus marmoratus* was considered in both studies. A phylogenetic analysis designed to study the relationships in the genus *Physalaemus*, however, is crucial to test this hypothesis. Also, further molecular characterization of the heterochromatic bands on 3p could help to provide additional evidence of the inferred heterochromatin amplification process.

In addition to the large C-band in 3p, the karyotype of *Physalaemus santafecinus* is also similar to those of *Physalaemus biligonigerus*, *Physalaemus marmoratus* and *Physalaemus* sp. aff. *biligonigerus* (Amaral et al. 2000, Silva et al. 2000) based on the presence of several telomeric C-bands and a pericentromeric C-band in the short arm of chromosome 8 as well as the NOR location. In all of these karyotypes, the NOR-bearing chromosome is small and metacentric, and it was classified as chromosome 9 in the karyotype of *Physalaemus santafecinus* (described here) and in the karyotypes of *Physalaemus biligonigerus*, *Physalaemus marmoratus* and *Physalaemus* sp. aff. *biligonigerus* described by Amaral et al. (2000). However, in the karyotype of *Physalaemus biligonigerus* described by Silva et al. (2000), the NOR-bearing chromosome was considered to be chromosome 8, which has a conspicuous pericentromeric C-band. Because Silva et al. (2000) apparently did not perform sequential C-banding and Ag-NOR in order to properly identify the NOR-bearing chromosome in C-banded metaphases, it is likely that the NOR-bearing chromosome is chromosome 9 in the C-banded karyotype shown by those authors.

In contrast to *Physalaemus santafecinus*, *Physalaemus biligonigerus*, *Physalaemus marmoratus* and *Physalaemus* sp. aff. *biligonigerus*, the telomeric C-bands could not be detected in the karyotype of *Physalaemus albifrons*. Additionally, the NOR in *Physalaemus albifrons* was detected interstitially in the long arm of the submetacentric chromosome 8. This NOR-bearing chromosome very closely resembles the NOR-bearing chromosome found in some populations of *Physalaemus cuvieri* (Silva et al. 1999, Quinderé et al. 2009) as well as in *Physalaemus albonotatus* and *Physalaemus cuqui* (present work). The *Physalaemus albifrons* karyotype presented here is very similar to the Giemsa-stained karyotype described for this species by Denaro (1972). However, the chromosome classified by Denaro (1972) as No. 11 is probably the one we classified as No. 8, and the secondary constriction observed by Denaro (1972) is likely to be the site recognized as NOR by silver impregnation in the present work.

Despite the similarity between the NOR-bearing chromosome of *Physalaemus albifrons* and those of some species of the *Physalaemus cuvieri* group, it would be premature to consider this a synapomorphy of *Physalaemus albifrons* and species of the *Physalaemus cuvieri* group because the evolutionary divergence of this character (i.e., NOR location) has not yet been elucidated. We cannot discard the possibility that the NOR found in *Physalaemus albifrons* and in some *Physalaemus cuvieri* species is plesiomorphic with respect to the other NOR sites found in *Physalaemus* species. This interpretation derives from the fact that the NOR-bearing chromosome 8 found in other leiuperines, as *Pleurodema diplolister* (Peters, 1870) (Lourenço et al., 2006), resembles that of *Physalaemus albifrons* and some *Physalaemus cuvieri* species group and could constitute the same state of character.

Another chromosome feature found in *Physalaemus albifrons* that was also detected in species of the *Physalaemus cuvieri* group was the interstitial C-band in chromosome 5 (Table 4). This C-band was observed in all of the species of the *Physalaemus cuvieri* group already analyzed by the C-banding technique, including *Physalaemus cuvieri* (Silva et al. 1999, Quinderé et al. 2009), *Physalaemus ephippifer* (Nascimento et al. 2010), *Physalaemus albonotatus* (present work), *Physalaemus centralis* (present work) and *Physalaemus cuqui* (present work). However, this band was not detected in the C-banded karyotypes of the other three species currently allocated in the *Physalaemus albifrons* group (Amaral et al. 2000, Silva et al. 2000, present work) or in species of the *Physalaemus henselii* group (Tomatis et al. 2009), the *Physalaemus olfersii* group (Milani et al. 2010) and the *Physalaemus gracilis* group (Provete et al. 2012). Based on these data, the interstitial C-band in the medium-sized chromosome classified as No. 5 is a putative synapomorphy of *Physalaemus albifrons* and the species of the *Physalaemus cuvieri* group. However, because of the small size of this C-band, which could make its detection by the C-banding technique particularly difficult, and because of the dynamics of the satellite DNA sequences, which are subject to recurrent amplification/deletion events, this hypothesis must be taken with caution. A comprehensive phylogenetic study of the genus *Physalaemus* and a molecular characterization of this interstitial C-band would allow this hypothesis to be properly evaluated.

**Table 4.** Comparison of chromosome 5 of species of *Physalaemus cuvieri* (left column) and *Physalaemus albifrons* (right column) groups. Black areas in the ideograms represent C-bands. *^1^C-band was tentatively assigned to the short arm (see text for details). *^2^Based on Silva et al. (1999) and Quinderé et al. (2009). *^3^Nascimento et al. (2010). *^4^Based on chromosomes described as No. 3 by Amaral et al. (2000).

***Physalaemus cuvieri* group (sensu Nascimento et al. 2005)**	**Chromosome 5**		***Physalaemus albifrons* group (sensu Nascimento et al. 2005)**
*Physalaemus albonotatus*	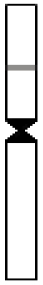 *^1^	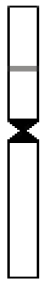	*Physalaemus albifrons*
*Physalaemus centralis*	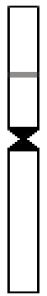	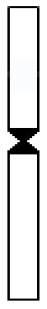 *^4^	*Physalaemus biligonigerus*
*Physalaemus cicada*	No C-banding data	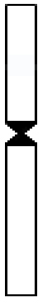 *^4^	*Physalaemus marmoratus* (=*Physalaemus fuscomaculatus*)
*Physalaemus cuqui*	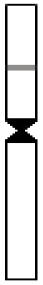	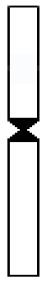	*Physalaemus santafecinus*
*Physalaemus cuvieri*	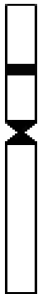 *^2^		
*Physalaemus ephippifer*	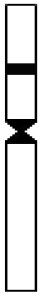 *^3^		
*Physalaemus erikae*	No C-banding data		
*Physalaemus fischeri*	No C-banding data		
*Physalaemus kroyeri*	No C-banding data		

In conclusion, we were not able to recognize any chromosomal character that would support the reallocation of *Physalaemus albifrons* from the *Physalaemus cuvieri* group to the *Physalaemus albifrons* group together with *Physalaemus biligonigerus*, *Physalaemus marmoratus* and *Physalaemus santafecinus*.

Interestingly, in addition to the data regarding chromosomal characteristics, larval morphology also does not seem to support the composition of the *Physalaemus albifrons* group. *Physalaemus biligonigerus*, *Physalaemus santafecinus* and *Physalaemus marmoratus* have a similar larval oral disc configuration (LTRF 2/2, with a dorsal gap in the marginal papillae) that differs considerably from that of *Physalaemus albifrons*, whose oral disc is almost identical to that of the tadpoles of the *Physalaemus cuvieri* group and is thus characterized by an LTRF 2/3 with dorsal, ventrolateral and ventral gaps in the marginal papillae (Vera Candioti et al. 2011). During embryogenesis of the oral disc of *Physalaemus*, ventrolateral gaps appear in the marginal papillae, apparently in all species of the genus (see Vera Candioti et al. 2011). The ventrolateral gaps persist only in the tadpoles of *Physalaemus cuvieri* species group [except *Physalaemus fischeri* (Boulenger, 1890) and *Physalaemus cicada* Bokermann, 1966], in *Physalaemus riograndensis* Milstead, 1960 (*Physalaemus henselii* group) and in *Physalaemus albifrons* (see Vera Candioti et al. 2011). On the other hand, ventral gaps develop only in tadpoles of *Physalaemus albifrons*, in species of *Physalaemus cuvieri* group (except *Physalaemus fischeri*) and in two species of the *Physalaemus henselii* group [*Physalaemus henselii* (Peters, 1872) and *Physalaemus fernandezae* (Müller, 1926)]. Among the leiuperines, the ventrolateral gaps were only observed in some species of *Pseudopaludicola* (see Vera Candioti et al. 2011), and although its presence during development appears to be plesiomorphic for *Physalaemus*, its persistence in larval stages is a putative synapomorphy of the *Physalaemus cuvieri* group (including *Physalaemus albifrons*). Finally, the internal oral morphology of tadpoles of *Physalaemus albifrons* differs from that of *Physalaemus biligonigerus*, *Physalaemus marmoratus* and *Physalaemus santafecinus* based on the presence of three lingual papillae, which is a characteristic shared with some species of the *Physalaemus cuvieri* group (Oliveira et al. 2010).

### Interspecific comparison in the *Physalaemus cuvieri* group

Some of the species in the *Physalaemus cuvieri* group are sibling species with important intraspecific morphological variation. Therefore, the identification of these species that is based exclusively on their morphology is sometimes very difficult. Occasionally, species misidentification has occurred, for example, among *Physalaemus cuvieri*, *Physalaemus albonotatus*, *Physalaemus cuqui* and *Physalaemus centralis* (Barrio, 1965). Our results revealed conspicuous cytogenetic differences among most species of the *Physalaemus cuvieri* group. The exception is the great similarity between the karyotypes of *Physalaemus albonotatus* and *Physalaemus cuqui*. Additionally, the karyotypes of the species analyzed here were distinguished from the previously analyzed karyotype of *Physalaemus cuvieri*. The interspecific variation described in this work regarding heterochromatin and NOR distribution is of fundamental importance for the comparative analysis of the *Physalaemus cuvieri* species group.

An interstitial C-band was observed near the centromere in the long arm of chromosome 2 of *Physalaemus albonotatus*, *Physalaemus centralis* and *Physalaemus cuqui*; whereas in the karyotype of *Physalaemus ephippifer* (Nascimento et al. 2010) there is an interstitial C-band in the short arm of chromosome 2. A corresponding interstitial C-band in the short arm of chromosome 2 was reported in *Physalaemus cuvieri* populations from Rio Claro (Silva et al. 1999) and Palmeiras (Quinderé et al. 2009). If these heterochromatic bands were homeologous, it is conceivable that rearrangements (mainly pericentric inversions) involving chromosome 2 might have occurred during the divergence of these species. Interestingly, the present work reports evidence of a rearrangement involving chromosome 2 in *Physalaemus centralis*. In the ZUEC 13696 specimen of *Physalaemus centralis*, heteromorphism for the intrachromosomal location of heterochromatic regions in the chromosome pair 2 suggested that paracentric inversion might have been involved in this chromosomal rearrangement.

Despite the overall similarity in chromosomal morphology among the species currently allocated to the *Physalaemus cuvieri* group, chromosome pairs 8 and 9 differ greatly. The differences in these chromosomes probably arose from the distinct locations of the NOR in these karyotypes, as these rDNA genes occupy different sites in pairs 8 and/or 9 of these species. The observed pattern of NOR occurrence can be helpful in distinguishing the analyzed species of the *Physalaemus cuvieri* group. Noticeably, a pericentromeric NOR site was found exclusively in the *Physalaemus centralis* karyotype. However, the NOR-bearing chromosomes (chromosome pairs 8) from the species *Physalaemus cuvieri* (Silva et al. 1999, Quinderé et al. 2009), *Physalaemus albonotatus*, *Physalaemus cuqui* and *Physalaemus albifrons* are quite similar and their homeology could be possible. Otherwise, the evolutionary relationship of this chromosome with the other NOR-bearing chromosomes found in species of *Physalaemus cuvieri*, *Physalaemus albifrons* and other species groups remains unclear, and further studies are necessary to elucidate the rearrangements that give rise to the great diversification of the NOR-bearing chromosomes in this genus.

## Figures and Tables

**Additional file 1.**
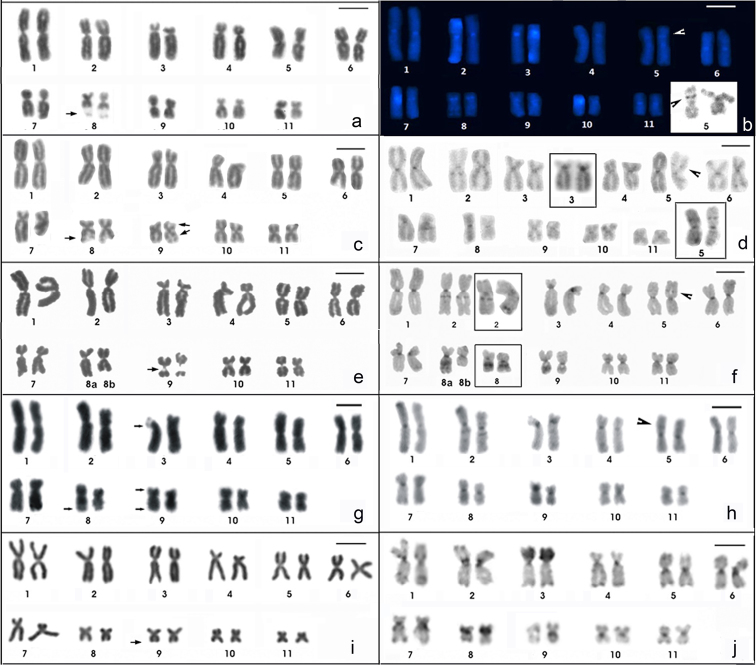
Giemsa-stained (**a, c, e, g,** **i**) and C-banded karyotypes of *Physalaemus albifrons* (**a,** **b**) *Physalaemus albonotatus* (**c, d**) *Physalaemus centralis* (**e, f**) *Physalaemus cuqui* (**g, h**) and *Physalaemus santafecinus* (**i, j**). In **b** the chromosomes were stained with DAPI after C-banding, except those in the inset, which were stained with Giemsa. In the insets in **d** C-banded chromosome pairs 3 and 5, showing evident pericentromeric and interstitial bands, respectively. The insets in **f** show the heteromorphic pair 2 and the homomorphic pair 8 of the ZUEC 13696 specimen. Arrows point NORs. Arrowheads point the C-band in 5p. Bar=10mm.
